# Brca1 Mutations Enhance Mouse Reproductive Functions by Increasing Responsiveness to Male-Derived Scent

**DOI:** 10.1371/journal.pone.0139013

**Published:** 2015-10-21

**Authors:** Ying Liu, Malcolm C. Pike, Nancy Wu, Yvonne G. Lin, Sara Mucowski, Vasu Punj, Yuan Tang, Hai-Yun Yen, Frank Z. Stanczyk, Elena Enbom, Theresa Austria, Martin Widschwendter, Robert Maxson, Louis Dubeau

**Affiliations:** 1 Department of Pathology, University of Southern California Los Angeles, Los Angeles, California, United States of America; 2 Department of Epidemiology and Biostatistics, Memorial Sloan Kettering Cancer Center, New York, New York, United States of America; 3 Department of Biochemistry and Molecular Biology, University of Southern California Los Angeles, Los Angeles, California, United States of America; 4 Department of Obstetrics and Gynecology, University of Southern California Los Angeles, Los Angeles, California, United States of America; 5 USC Norris Comprehensive Cancer Center Bioinformatics Core and Division of Hematology, University of Southern California Los Angeles, Los Angeles, California, United States of America; 6 Department of Women’s Cancer, University College, London, United Kingdom; CNR, ITALY

## Abstract

We compared the gene expression profiles of ovarian granulosa cells harboring either mutant or wild type *Brca1* to follow up on our earlier observation that absence of a functional Brca1 in these important regulators of menstrual/estrous cycle progression leads to prolongation of the pre-ovulatory phase of the estrous cycle and to increased basal levels of circulating estradiol. Here we show that ovarian granulosa cells from mice carrying a conditional *Brca1* gene knockout express substantially higher levels of olfactory receptor mRNA than granulosa cells from wild type littermates. This led us to hypothesize that reproductive functions in mutant female mice might be more sensitive to male-derived scent than in wild type female mice. Indeed, it is well established that isolation from males leads to complete cessation of mouse estrous cycle activity while exposure to olfactory receptor ligands present in male urine leads to resumption of such activity. We found that *Brca1* ^*-/-*^ female mice rendered anovulatory by unisexual isolation resumed ovulatory activity more rapidly than their wild type littermates when exposed to bedding from cages where males had been housed. The prime mediator of this increased responsiveness appears to be the ovary and not olfactory neurons. This conclusion is supported by the fact that wild type mice in which endogenous ovaries had been replaced by *Brca1*-deficient ovarian transplants responded to male-derived scent more robustly than mutant mice in which ovaries had been replaced by wild type ovarian transplants. Our findings not only have important implications for our understanding of the influence of olfactory signals on reproductive functions, but also provide insights into mechanisms whereby genetic risk factors for breast and extra uterine Müllerian carcinomas may influence menstrual activity in human, which is itself an independent risk factor for these cancers.

## Introduction

The high-grade serous subtype is the most common and the deadliest histological subtype of extra uterine Müllerian carcinomas. Germline *BRCA1* mutations are the greatest risk factor for the familial form of these tumors, while menstrual cycle activity is the greatest known risk factor for the sporadic form [1–3]. We previously tested the hypothesis that these genetic and reproductive risk factors were interrelated using a rodent model in which *Brca1*, the homolog of human *BRCA1*, was knocked out in granulosa cells, which are the major steroid producing cells of the ovary, and in the anterior pituitary, which controls steroidogenesis in granulosa cells via secretion of gonadotropin hormones [4,5]. Mutant mice, although fertile, showed an elongation of the proestrus phase of their estrous cycle (corresponding to the pre-ovulatory phase of the human menstrual cycle) relative to the metestrus phase (corresponding to the human post-ovulatory phase), resulting in increased duration of unopposed estrogen stimulation [5]. The mutant mice also showed elevation of circulating estradiol levels [5]. Ovarian granulosa cells drove these hormonal changes independently of the pituitary gland [5]. Subsequent observations showed increased endometrial cell proliferation and increased bone length and density in mutant mice, all of which are established consequences of increased estrogen stimulation [6]. The relevance of these findings to human *BRCA1* mutation carriers is underscored by our recent findings of alterations in sex steroid hormone levels and in endometrial thickness in *BRCA1* mutation carriers compared to non-carriers [7].

We used microarray technologies to compare the gene expression profiles of mutant and wild type granulosa cells in our experimental model in order to gain insight into mechanisms whereby Brca1 may influence menstrual/estrous cycle regulation. Here we report that genes belonging to the olfactory receptor family are among those showing the greatest degree of differential expression in ovaries of mutant relative to wild type mice. We then took advantage of the well-established phenomenon whereby the estrous cycle of mice rendered anovulatory by unisexual isolation resumes in the presence of male-derived scent, such as that present in bedding from cages where males have been housed [8–10], to test the hypothesis that olfactory influences on reproductive functions are quicker in mutant mice and to investigate the role of the ovary, relative to that of the central nervous system, in mediating such influence.

## Materials and Methods

### Ethics statement

All studies with human subjects were approved by the Institutional Review Board of the University of Southern California. Participating human subjects were only enrolled after they signed an appropriate consent form approved by the Institutional Review Board. All studies with experimental animals were approved by and performed under supervision of the University of Southern California Institutional Animal Care and Use Committee.

### Source and handling of experimental animals

The generation of *Fshr-Cre; Brca1* ^*flox/flox*^ mice was described earlier [4,5]. Animals were housed in a pathogen-free environment at the Vivaria facility of the USC Health Sciences campus. For studies where females needed to be isolated from males, we used a separate and fully functional mouse facility where other mice were not being housed at that time. All facilities, including the latter, received daily monitoring and care from Vivaria staff under the supervision of a veterinarian. A maximum of 4 mice were housed in each cage. Assignment to each experimental group was based on genotype. For studies examining response to male scent, mice of different genotypes were housed in the same cages in order to ensure equal exposure to male-derived scent. Observations about estrous cycle activity were scored without knowledge of genotype in order to avoid observer bias. Procedures for performance of ovarian transplantations and for evaluation of estrous cycle activity were described earlier [5]. Euthanasia was achieved by cervical dislocation after the mice were made unconscious from exposure to CO_2_.

### Expression profiling analyses

A total of 4 mice were used, including 2 mutant and 2 wild type. For each genotype, one mouse was 8 months old and the other was 11 months old. The mice were inoculated with 5 IU of pregnant mare serum gonadotropins (PMSG, Sigma, St. Louis, MO; catalog #G4877) in order to synchronize them into the proestrus phase of their estrous cycle and were euthanized exactly 48 hours later. Both ovaries were collected and pooled from each mouse. The ovaries were frozen in OCT blocks, from which 5 micron-thick histological sections were obtained. Granulosa cells were isolated by laser capture microdissection. Libraries of cDNA synthesized from mRNA extracted from the microdissected granulosa cells were hybridized to exon array Mouse Exon 1.0 St Array chips (Affymetrix, San Diego, CA), which comprehensively represents all the exons in the entire genome. The chip was washed and scanned with the Gene Chip Scanner 3000. After background correction, data analysis was done using RMA (Robust Multichip Average expression) algorithm. Transcript level data were summarized to obtain differential expression among wild type and mutant cells at the gene level.

### Quantitative RT-PCR studies

Quantitative RT-PCR analyses for calculation of Pearson correlation coefficient and for comparing olfactory receptor levels between homozygous, heterozygous, and wild type mice were performed using mRNA extracted from microdissected granulosa cells obtained from both ovaries of 3 wild type mice and 3 mutant mice synchronized into the proestrus phase. All mice were 8 months old. We used a TaqMan 7900 instrument (Applied Biosystems, Carlsbad, CA). We used the following primer sequences for these respective loci: 5'-ATGTTCTTGGAAATGCTGAACCC-3' (forward) and 5'-AGGACCTGGTATTGAAGACGAG-3' (reverse) for amplification of *Cyp19a1*; 5'-AGGACCTGGTATTGAAGACGAG-3' (forward) and 5'-GCCCAAGTCAAAGACACCTAAT -3' (reverse) for amplification of *Cyp17a1*; 5'-AGTGCTAAATAGCGTGTTTACCA-3' (forward) and 5'-ACTTTTTGTGTAGTGTCTCCCTG-3' (reverse) for amplification of *Hsd3b*; 5'-ACTTGGCTGTTCGCCTAGC-3' (forward) and 5'-GAGGGCATCCTTGAGTCCTG-3' (reverse) for amplification of *Hsd17b1*; 5'-AAATCACCATGCCCTCTACAAG-3' (forward) and 5'-CCCACTTTTATCACCATCGCAA-3' (reverse) for amplification of *S100a8*; 5'-TGCAGGTTGTGTGTCCCAG-3' (forward) and 5'-TGAGTAGCGTAGAGGGTAGCA-3' (reverse) for amplification of *Olfr149*; 5'-AGAAACCGCAGCTCTCTTACC-3' (forward) and 5'-GGCGGGAATCCATGTGTATCA-3' (reverse) for amplification of *Olfr62*; 5'- CTTCCTGGCTTTACTTTCCTTCA-3' (forward) and 5'- CCAGACTCCAGGCCAGTCAACA-3' (reverse) for amplification of *Olfr666*; 5'-ACAATCTCACGAATGGATCAGC-3' (forward) and 5'-CACCCAGCATAGCTCATGGT-3' (reverse) for amplification of *Olfr1383*; 5'-AGGTCCTTCAATGAGATCCCTT-3' (forward) and 5'-TCCCTGTAAATGGGGCCATAC-3' (reverse) for amplification of *Cyp11a1*; 5'-GACCCCTTTGTGTGATGTATGC-3' (forward) and 5'-GACTAGACAGGCGTCAAAAGC-3' (reverse) for amplification of *Olfr641*; 5'-TCTTGCAGGCTTGACAGATGC-3' (forward) and 5'-CCTTGGGGGTGATTGCTGAG-3' (reverse) for amplification of *Olfr1471*; 5'-GCACAGTCAAGGCCGAGAAT-3' (forward) and 5'-GCCTTCTCCATGGTGGTGAA-3' (reverse) for amplification of *Gapdh*. Real-time detection of the amplification products was achieved using a SYBR super mix kit (Fermentas, Pittsburgh, PA, catalog #4309155) and normalized against *Gapdh*.

### Collection of human granulosa cells

Human granulosa cells were obtained from overages of *in vitro* fertilization procedures after approval by the Institutional Review Board of the University of Southern California. Follicular fluid was obtained at the time of oocyte aspiration and the eggs were removed after microscopic inspection. Granulosa cells suspended in follicular fluid were transported at room temperature to the laboratory. After addition of an equal volume of Ficoll reagent, the samples were centrifuged at 400 x G for 20 minutes. The granulosa cell layer was washed twice in PBS, resuspended in 1 mL of this buffer, and kept frozen at -80°C until used for RNA or protein extraction.

### Establishment of human granulosa cell primary cultures

Luteinized granulosa cells were obtained from volunteers undergoing controlled ovarian hyperstimulation for *in vitro* fertilization at the USC Fertility Center after approval from our Institutional Review Board. Cells transported in follicular fluid were centrifuged at 1000 x G for 10 minutes. For each 0.5 mL of pellet volume, 9 mL of sterile water was added for exactly 20 seconds, followed by addition of 1 mL of 10X PBS. After centrifugation, the cells were plated in 50% DMEM and 50% F12 supplemented with 5% horse serum, 33% follicular fluid, 80 microM ascorbic acid, 0.05 microM dexamethasone, 20 ng/mL EGF, 50 ng/mL beta-FGF, 1 ng/mL follicle stimulating hormone, 50 microgram/mL gentamycin, and 1% Penicillin/Streptomycin. The concentration of follicular fluid was gradually decreased until totally omitted over a period of one week.

### Immunohistochemistry

Anti-Olfr68 (Abcam, Cambridge, MA, catalog #ab62606), anti-Olfr1508 (Abcam, catalog #ab65734), and anti-adenylyl cyclase 3 (Santa Cruz Biotechnology, catalog #sc-588) antibodies were used at a dilution of 1:100 on formalin-fixed, paraffin-embedded tissue sections. After deparaffinization, endogenous peroxidase activity was inactivated with 3% hydrogen peroxide for 10 minutes. Normal goat serum was added as blocking agent for 1 hour, followed by an overnight incubation at 4°C with primary antibody and a 30-minute incubation with biotinylated goat anti-rabbit antibody provided with the ImmunoCruz rabbit ABC Staining System (Santa Cruz Biotechnology, catalog #sc2018). Slides were counterstained with hematoxylin.

### Western blot analyses

Total cellular protein extracts were obtained from homogenized mouse olfactory tubercles, mouse ovaries, and human luteinized granulosa cells from donors undergoing *in vitro* fertilization procedures. The following denaturation conditions were needed in order to achieve reduction of the oligomeric forms of olfactory receptor proteins into monomers: protein extracts in buffer supplemented with 3% sodium dodecyl sulfate and 0.5M freshly prepared dithiothreitol were incubated for 10 minutes in boiling water. Samples were electrophoresed on 10% acrylamide– 0.1% SDS gels and transferred to PVDF membranes (Bio-Rad, Hercules, CA, catalog #1620177), followed by hybridization to rabbit polyclonal Olfr68 (Abcam, catalog #ab65467) antibody overnight at 4°C. Hybridization of secondary antibody (horse radish peroxidase conjugated anti-rabbit, Santa Cruz Biotechnology, catalog #sc2004) was for 1 hour at room temperature. Dilutions for the primary and secondary antibodies were 1:200 and 1:2500, respectively. Hybridization signals were visualized using Pierce ECL Western Blotting Substrate (Thermo Fisher, Waltham, MA, catalog #32109).

### Source of glycosidases

Endoglycosidase H and Peptide N-glycosidase F were obtained from New England Biolabs (Ipswich, MA, catalog #P0702S and P0704S, respectively).

### Statistical methods

Fisher’s exact test was used to determine the significance of differences in the proportion of mice from different genotypes that had resumed estrous cycle activity at pre-determined time points. The exact conditional combined test was used to look at the overall significance of such differences in multiple experiments. Differences in olfactory receptor mRNA expression between granulosa cells of wild type, heterozygous, and homozygous *Brca1* mutant mice were evaluated for their statistical significance using Student *t*-test.

## Results

### Up-regulation of olfactory receptors in ovarian granulosa cells lacking a functional Brca1

**Fig 1:**
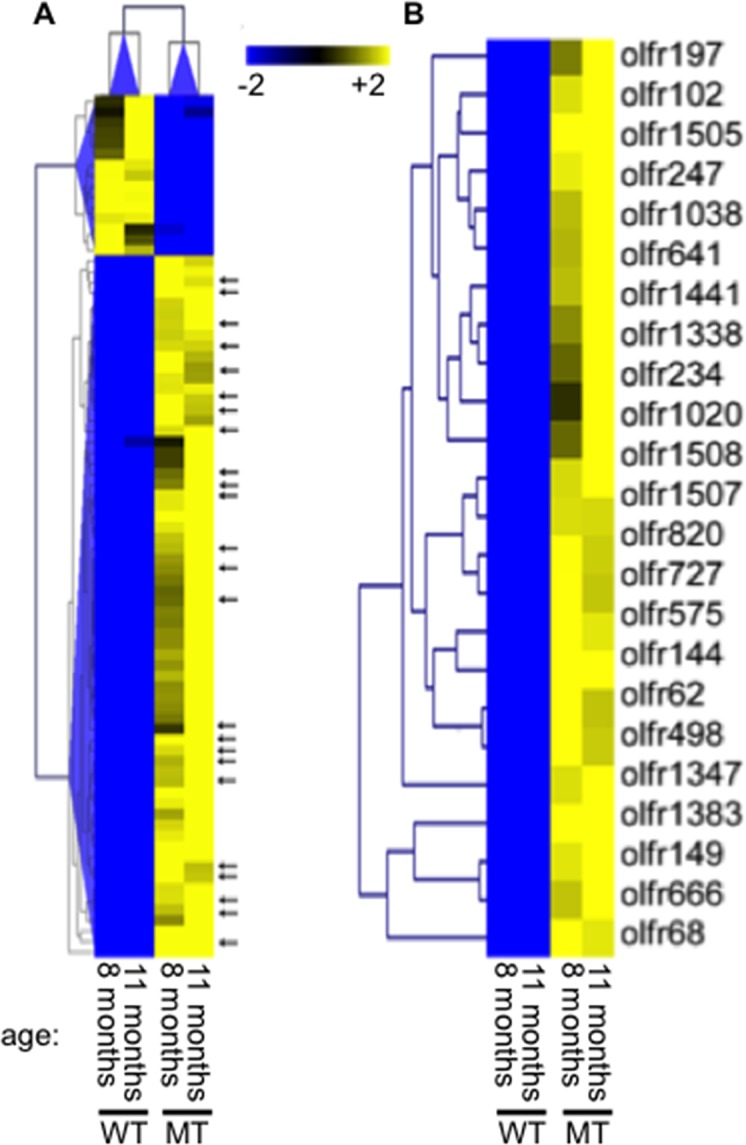
Increased expression of olfactory receptors in ovarian follicles lacking a functional Brca1. Two pairs of wild type (WT) and mutant (MT) mice, respectively 8 and 11 months old, were synchronized in the proestrus phase of their estrous cycle. Granulosa cells were isolated from frozen histological sections of their ovaries using laser capture microdissection. Expression profiling analyses were performed using total mRNA from each cell preparation. (A) Heat map illustrating the expression levels of genes showing differential down-regulation (blue) or up-regulation (yellow) of at least 2-fold in mutant mice from both age groups. The arrows indicate genes belonging to the olfactory receptor family. The heat map in (B) shows olfactory receptor genes with at least 2-fold differential expression in both age groups.

**Fig 2:**
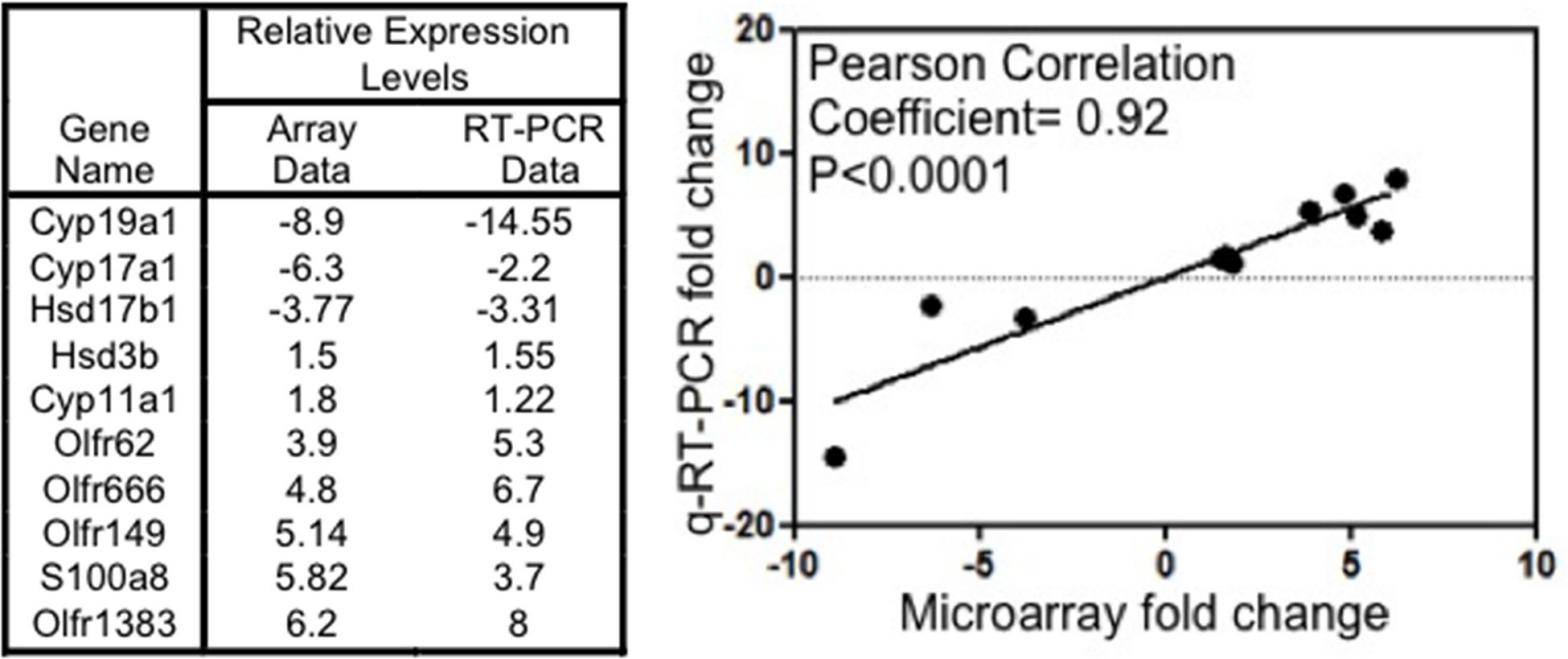
Correlation between gene expression levels determined from microarray *versus* RT-PCR analyses. Ten loci were randomly selected from the list of differentially expressed loci in S1 Table and their relative expression levels were measured using quantitative RT-PCR. Relative expression values obtained from the expression profiling studies and from the RT-PCR data are shown in the table on the left and plotted in the graph shown on the right. The Pearson correlation coefficient was calculated to evaluate the correlation between expression array and real time RT-PCR data.

Granulosa cells were microdissected from ovaries of *Fshr-Cre; Brca1* ^*flox/flox*^ mice lacking a functional Brca1 and from ovaries of wild type littermate controls. The mice were inoculated with pregnant mare serum gonadotropins 48 hours prior to being euthanized to synchronize their estrous cycle into the pre-ovulatory (proestrus) phase corresponding to the human follicular phase. The gene expression profiles of mutant and wild type ovaries were compared in 2 independent studies using mice of 2 different ages (8 months old and 11 months old, respectively). A complete list of genes up- or down-regulated at least 2-fold in both mutant animals relative to their age-matched wild type littermates is provided as supporting information (S1 Table). A hierarchical clustering of genes using Euclidean distance and average matrix showed distinct separation into wild type and mutant groups (Fig 1). Several of the genes that were upregulated in both mutant animals belonged to the olfactory receptor family, indicated by arrows in the heat map shown in Fig 1A. A separate heat map focused specifically on olfactory receptor loci meeting the criteria of being differentially regulated at least 2-fold in both mutant animals is shown in Fig 1B. Analysis of randomly selected loci by quantitative RT-PCR in microdissected granulosa cells from mutant and wild type animals showed excellent correlation (Pearson correlation coefficient = 0.92, P<0.0001) between the gene expression profiling and the RT-PCR data (Fig 2).

**Fig 3:**
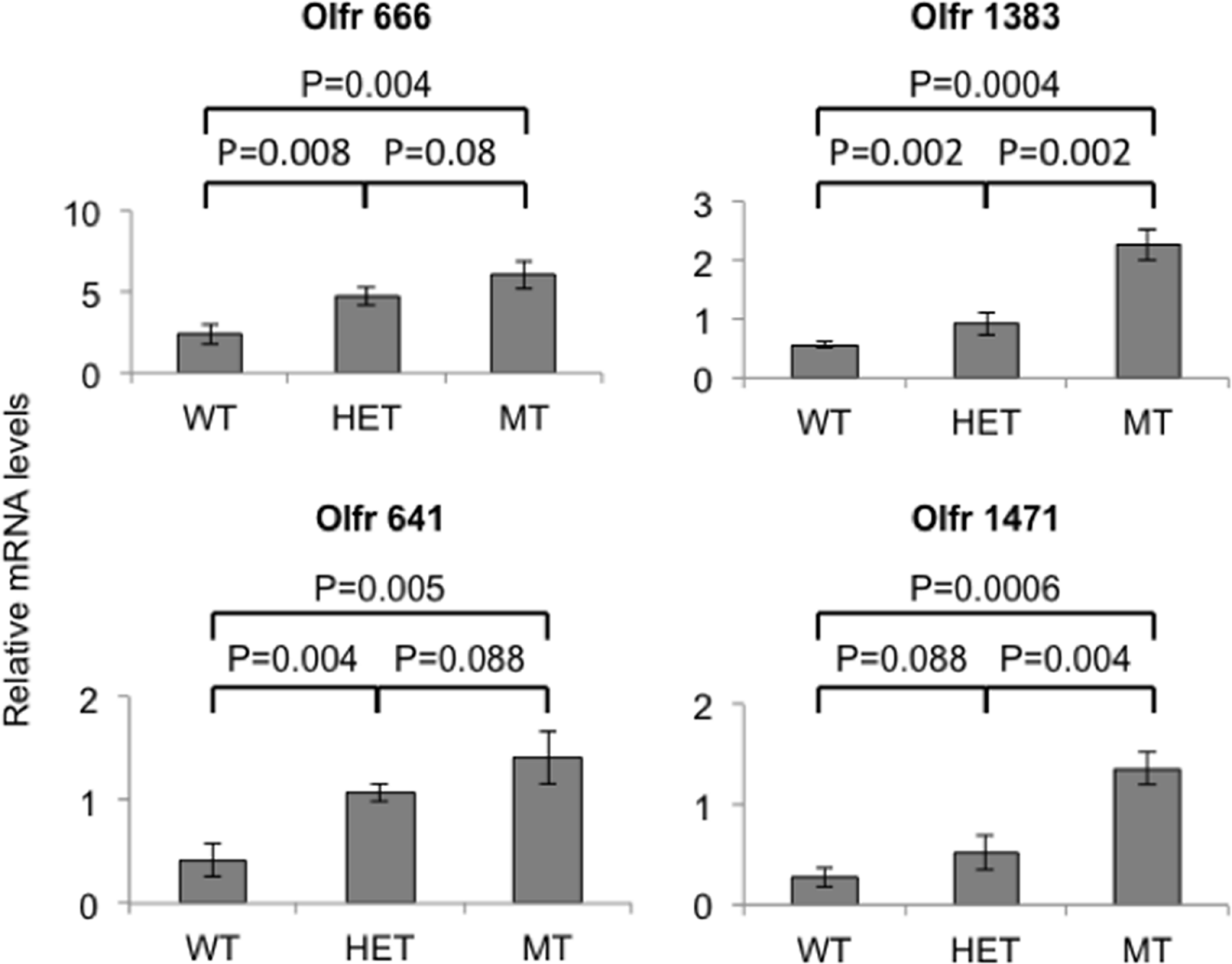
Levels of olfactory receptor mRNAs in mice carrying a heterozygous *versus* homozygous conditional *Brca1* gene knockout. Total mRNA isolated from microdissected granulosa cells from the ovaries of 3 different *Fshr-Cre; Brca1* ^*+/+*^ mice (WT), 3 different *Fshr-Cre; Brca1* ^*flox/+*^ mice (HET), and 3 different *Fshr-Cre; Brca1* ^*flox/flox*^ mice (MT) was subjected to quantitative real-time RT-PCR using primers for the indicated loci and a SYBR super mix kit on an ABI 7900 TaqMan instrument (Applied Biosystems). Relative mRNA levels for each gene of interest were normalized to those of an internal reference gene (*Gapdh*). The bar graphs represent means of the triplicates from each set ± standard deviation. Two-sided P values were calculated using Student’s *t*-test.

Human *BRCA1* mutation carriers invariably show heterozygous mutations in their germline. In humans, inactivation of both *BRCA1* alleles is present only in epithelial tumors, which are not derived from granulosa cells [11]. We used mice carrying a homozygous *Brca1* mutation in their granulosa cells in our studies in order to maximize the effects of such mutation, reasoning that the changes in *Brca1* gene dosage associated with a heterozygous mutation should lead to consequences similar to those seen in homozygous mutants, albeit of lesser magnitude. Indeed, mice carrying a heterozygous *Brca1* mutation in their ovarian granulosa cells showed increased olfactory receptor expression in these cells compared to wild type mice (Fig 3). Of the 4 olfactory receptor proteins examined in this study, 3 showed a statistically significant increase in heterozygous mice while the fourth one showed an increase of borderline statistical significance. In each case, the expression levels were intermediate between those seen in wild type animals and those seen in homozygous mutants.

### Confirmation of olfactory receptor expression in mouse and human ovarian granulosa cells

**Fig 4:**
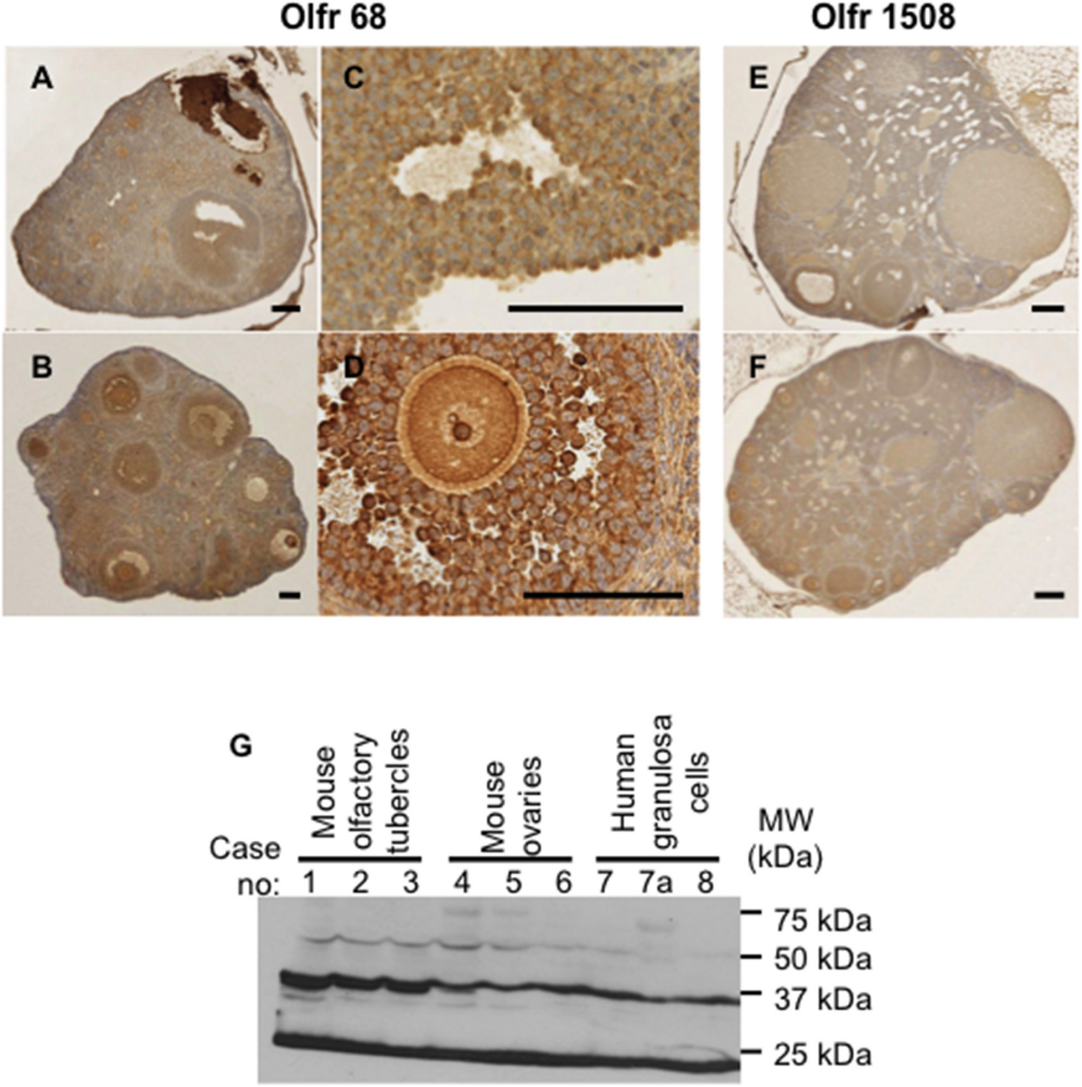
Olfactory receptor protein expression in mouse and human ovarian granulosa cells. Immunohistochemical stains of ovaries from wild type (A, C, E) and mutant (B, D, F) mice using either anti-Olfr68 (A-D) or anti-Olfr1508 (E-F). The high magnification images shown in C and D are from the same ovaries shown at lower magnification in A and B, respectively. All mice were littermates and were treated with 5 IU of PMSG 48 hours before being euthanized to mimic the conditions used for the mRNA profiling analyses. Bars: 100 microns. (G) Western blot analyses of protein extracts obtained from 3 different wild type mouse olfactory tubercles (samples 1–3), 3 different wild type mouse ovarian granulosa cells (samples 4–6), and 2 different normal donors of human luteinized granulosa cells undergoing *in vitro* fertilization procedures (samples 7–8). Cells from the donor from whom sample 7 was derived were also cultured *in vitro* for 8 days before being analyzed by western blotting (sample 7a). The blot was probed with an antibody against Olfr68, which shows 82% sequence homology to human OR52A5 and 73% homology to human OR52A1, both with molecular sizes of 36 KDa.

Immunohistochemical staining of ovaries from 2 sets of wild type and mutant mice, one with an antibody specific for Olfr68 and the other with an antibody specific for Olfr1508, two of the differentially expressed receptors between mutant and wild type animals in our expression profiling studies, are shown in Fig 4. We were unable to find a tissue type that was totally negative with this antibody, either based on immunohistochemistry or western blotting analyses in accordance with earlier reports that olfactory receptors are widely expressed in most, if not all organs [12,13]. Nevertheless, the staining intensity of ovarian follicles is clearly greater than that of ovarian stroma (Fig 4) in support of the hypothesis that these structures are an important site of olfactory receptor signaling within the ovary. Although differences in the staining intensity between wild type and mutant ovaries must be interpreted with caution given the semi-quantitative nature of immunohistochemistry, the results show slightly increased intensity in mutant mice in support of our gene expression profiling studies. Examination of a Graafian follicle at higher magnification (Fig 4C and 4D) showed cytoplasmic and surface immunoreactivity, consistent with the notion that a substantial fraction of such receptors typically remains unprocessed in the endoplasmic reticulum [14]. We next analyzed protein extracts of mouse olfactory tubercle (positive control) and of ovaries by western blotting using an antibody against Olfr68. The results showed a predominant signal corresponding to the expected size of approximately 36 kDa (Fig 4G). Faint signals were also seen corresponding to proteins of 50 and 70 kDa. The intensity of the 70 kDa fragment was markedly increased when less stringent denaturing conditions were used prior to electrophoresis, suggesting that it represents a dimeric form of the olfactory receptor protein. Such dimerization is common among olfactory receptors, which belong to the G protein-coupled receptor family [15,16], and is thought to play important physiological roles [17]. We considered the possibility that the 50 kDa fragment represents a glycosylated product [18]. However, this fragment remained unchanged after digestion with either peptide N-glycosidase F or endoglycosidase H and its exact nature therefore remains unclear.

Fig 4G also shows western blot analyses of human granulosa cells probed with the Olfr68 antibody (samples 7, 7a, and 8). Olfr68 shows 82% sequence homology to human OR52A5 and 73% homology to human OR52A1, both with molecular sizes of approximately 36 kDa. The cells were collected from 2 different granulosa cell donors undergoing *in vitro* fertilization procedures and immediately subjected to protein extraction procedures without further manipulation (samples 7 and 8). Cells from sample 7 were also placed in tissue culture dishes and incubated for 8 days without passaging in medium supplemented with growth factors, horse serum and follicular fluid (sample 7a). A prominent signal of the expected size was obtained with all extracts from human cells, including those that were subjected to *in vitro* culturing, indicating that an antigen showing specific affinity for anti-Olfr68 not only is expressed by human granulosa cells, but also that such expression persists in short-term primary cultures.

### Increased response of mice carrying a *Brca1* mutation to male-derived scent

The results thus far show that olfactory receptors are expressed in ovarian granulosa cells and that the magnitude of expression is higher, at least at the RNA level, in the absence of a functional Brca1 protein. This led us to hypothesize not only that interactions between such receptors and their specific ligands might influence reproductive functions, but also that responses to such ligands might be increased in *BRCA1* mutation carriers. The influence of male-derived scent, including response to pheromones, on female reproductive functions has been well established in rodents. Female mice show a gradual decrease and, eventually, a complete cessation of estrous cycle activity following isolation from males [8]. Their cycle rapidly resumes upon re-exposure to male-derived scent [9,10]. An olfactory receptor ligand that mediates these effects has been isolated in the urine of male mice [19].

We investigated whether response to male scent is more pronounced in Brca1-deficient mice than in their wild type littermates. Mutant and wild type female mice were kept in total isolation from males in a building separate from our main Vivaria facility. Complete cessation of estrous cycle activity resulting from such unisexual isolation was documented by daily assessment of the appearance of the vaginal mucosa as previously described [20]. Bedding from cages housing male mice was then obtained and evenly admixed with bedding of cages housing the non-cycling female mice. Each cage contained a mixed population of mutant and wild type animals to ensure equal exposure to male-derived scent regardless of genotype. Furthermore, all examinations were performed without knowledge of the genotype until the findings were recorded in order to avoid observer bias. There were no noticeable differences in the overall well-being and external phenotypic characteristics of mutant *versus* wild type animals except for a slight increase in height in mutant mice reported earlier [6]. The vaginal mucosa of each mouse was examined for signs of resumption of estrous cycle activity every 3 hours. If such signs were seen, vaginal fluid was collected, spread on a glass slide, stained with Papanicolaou reagents, and examined microscopically to confirm resumption of cycling activity as described previously [5]. Of 11 mutant mice, 82% had resumed cycling activity 6 hours after exposure to male scent and 91% after 9 hours. In contrast, only 30% and 40% of 10 wild type mice had resumed such activity at these respective time points. All mice had resumed cycling activity 24 hours after exposure. We repeated this experiment focusing on the 9-hour time point. A higher number of mutant mice had resumed cycling activity at this time point compared to wild type animals in three independent experiments (Table 1).

**Table 1:** Differences in response to male-derived scent between wild type and mutant mice.

Study number	Time after exposure to male bedding	Genotype	Number of cycling mice	Number of non-cycling mice	P_(Fisher’s exact test)_ *
1	9 hours	wild type	4 (40.0%)	6 (60.0%)	0.024
	9 hours	mutant	10 (90.9%)	1 (9.1%)	
2	9 hours	wild type	2 (15.4%)	11 (84.6%)	0.022
	9 hours	mutant	5 (71.4%)	2 (28.6%)	
3	9 hours	wild type	1 (12.5%)	7 (87.5%)	0.041
	9 hours	mutant	6 (75.0%)	2 (25.0%)	

*Difference between genotypes for each study number.

Exact conditional combined test: P = 0.0001.

### Differential role of ovarian *versus* neuronal olfactory receptors in mediating a response to male-derived scent

**Fig 5:**
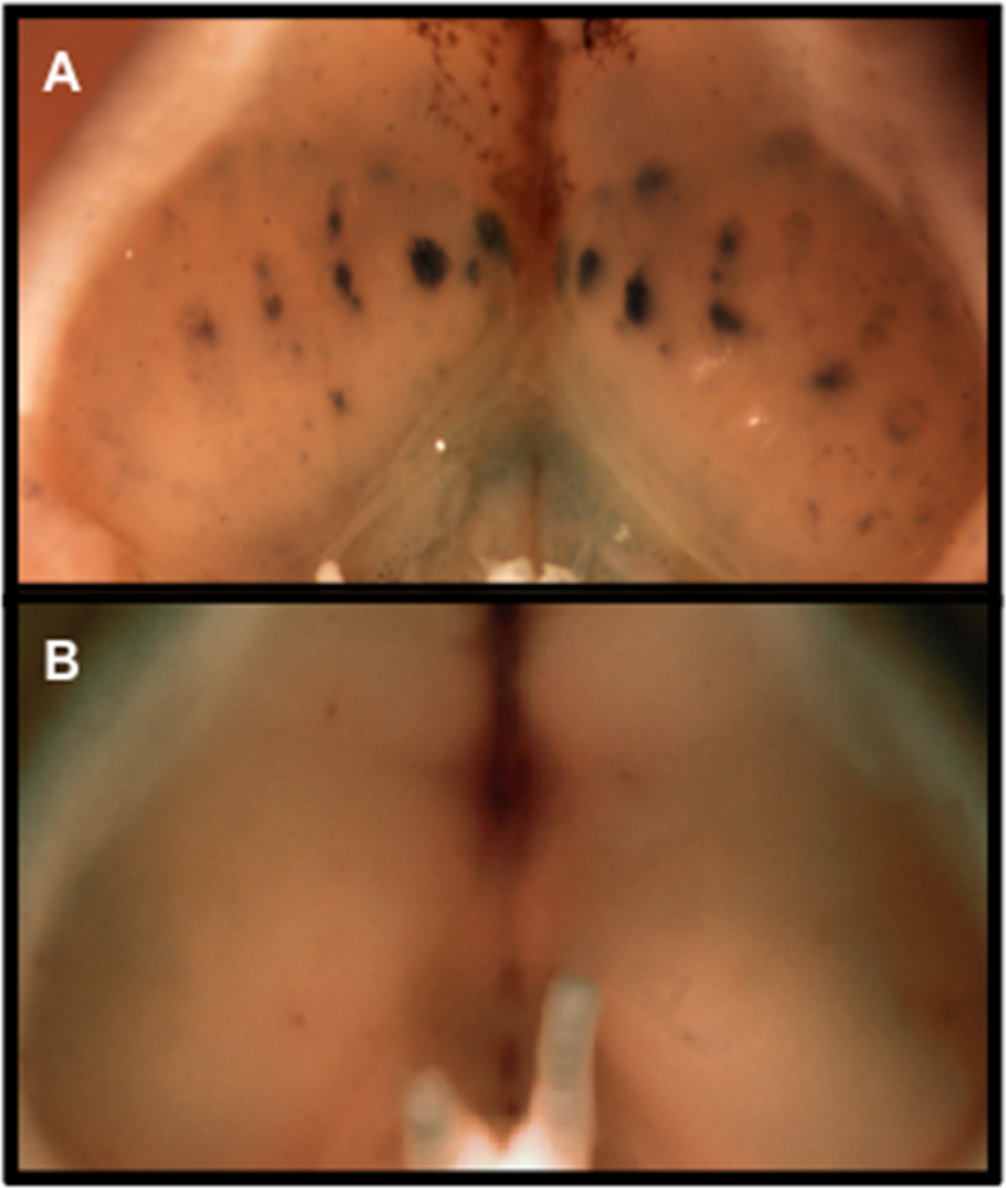
Cre-mediated recombination in olfactory tubercles of *Fshr-Cre* transgenic mice. Olfactory tubercles of R26R reporter mice either carrying (A) or not carrying (B) the *Fshr-Cre* transgene were stained for LacZ as reported earlier [4]. The blue color indicative of a positive LacZ colorimetric assay, present in olfactory bulbs from the transgenic line, indicates the presence of Cre-mediated recombination driven by the *Fshr-Cre* transgene in olfactory tubercles of mice harboring this transgene.

Our finding of an association between the presence of an ovarian-specific *Brca1* mutation and increased responsiveness of female rodents to male-derived scent raised the intriguing possibility that olfactory receptors present in the ovary, as opposed to the central nervous system, played a role in mediating this effect. However, the possibility remained that the cell-specific promoter used to drive Cre-mediated *Brca1* recombination in our experimental model is active in parts of the central nervous system responsive to olfactory stimuli. Indeed, transferring this promoter construct to the R26R reporter strain showed that it is active in the olfactory bulbs (Fig 5). We therefore generated mice in which *Brca1* was inactivated either in ovaries or in the central nervous system, but never in both organs simultaneously in order to evaluate their relative importance in mediating response to male-derived olfactory stimuli. This was achieved by transplanting ovaries of mutant mice under the renal capsule of previously oophorectomized wild type animals and also performing the reciprocal procedure where the ovaries of wild type animals were transplanted under the renal capsule of oophorectomized mutant mice. Confirmation that the transplanted ovaries remained functional was obtained by documentation of estrous cycle activity from cytological examination of vaginal lavages as described previously [5].

We first performed a study in which 24 wild type animals harboring renal sub-capsular ovarian transplants obtained from 24 wild type littermate donors were isolated from males until cessation of estrous cycle activity as in the previous study. The number of animals that had resumed cycling activity 9 hours after exposure to male-derived scent was then measured in order to verify that a response to such olfactory stimuli would be present in mice harboring transplanted ovaries similarly to what had been observed in mice with intact endogenous ovaries. Not only was such a response seen in transplant recipients, but also it appeared to be stronger than in mice with intact endogenous ovaries. Sixteen of 24 wild type mice harboring transplanted wild type ovaries had resumed ovulatory activity at this time point compared to 7 of 24 wild type mice not harboring any ovarian transplant (P_Fisher’s exact test_ = 0.02). We conclude that ovaries transplanted under the renal capsule responded faster to male-derived scent than endogenous ovaries, perhaps due to the larger fraction of the cardiac output being delivered to the kidneys.

We next compared the rapidity of response to male-derived scent in wild type mice harboring mutant ovaries to that in mutant mice harboring wild type ovarian transplants. As in the previous study, mice of different genotypes were admixed in the same cages and the genotypes were not known to the observer until the results had been recorded. The proportion of mice that had resumed cycling activity in each group was measured 3 hours after exposure to male-derived scent in order to account for the apparently more rapid response seen in mice harboring renal sub-capsular ovarian transplants. The proportion of wild type mice harboring mutant ovaries that had resumed cycling activity at this time point was consistently greater than the proportion of mutant mice harboring wild type ovaries (Table 2).

**Table 2:** Influence of ovarian genotype on response to male-derived scent.

Study number	Time after exposure to male bedding	Genotype	Number of cycling mice	Number of non-cycling mice	P_(Fisher’s exact test)_ *
1	3 hours	mouse^wt^; ovary^mt^	8 (57.1%)	6 (42.9%)	0.051
	3 hours	mouse^mt^; ovary^wt^	2 (16.7%)	10 (83.3%)	
2	3 hours	mouse^wt^; ovary^mt^	11 (78.6%)	3 (21.4%)	0.016
	3 hours	mouse^mt^; ovary^wt^	3 (25.0%)	9 (75.0%)	
3	3 hours	mouse^wt^; ovary^mt^	8 (88.9%)	1 (11.1%)	0.020
	3 hours	mouse^mt^; ovary^wt^	3 (30.0%)	7 (70.0%)	

*Difference between genotypes for each study number.

Exact conditional combined test: P < 0.0001.

mouse^wt^; ovary^mt^: wild type mouse with ovaries from a mutant donor.

mouse^mt^; ovary^wt^: mutant mouse with ovaries from a wild type donor.

### Evidence for intra-ovarian G protein-coupled receptor signaling

**Fig 6:**
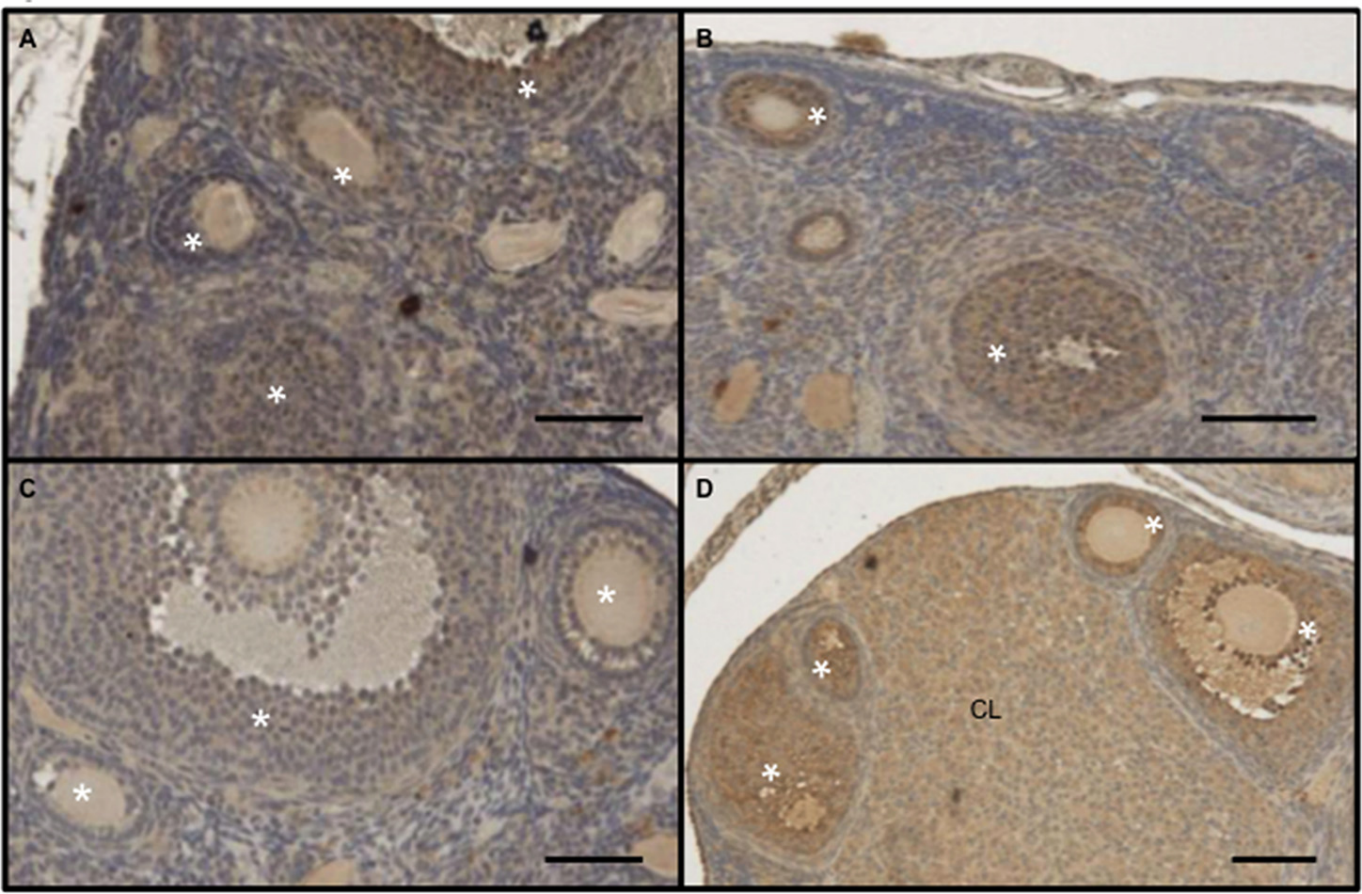
Increased expression of intra-ovarian adenylyl cyclase 3 following exposure to male-derived scent. Ovaries of 2 wild type (A, B) and 2 mutant (C, D) unisexually isolated mice, either unexposed (A, C) or exposed (B, D) to male-derived scent for 9 hours, were stained with a polyclonal antibody against adenylyl cyclase 3. Absence of estrous activity in mice not exposed to male-derived scent and presence of such activity in mice exposed to such scent were confirmed by vaginal cytology obtained just before the mice were euthanized. Stars are over the granulosa cell layers of ovarian follicles. CL: corpus luteum. Bars: 50 microns.

Olfactory receptors belong to the G protein-coupled receptor family, which typically activates adenylyl cyclase 3 upon ligand binding [21]. We therefore sought to determine whether this cyclase is present in ovarian granulosa cells and to investigate the influence of exposure to male derived scent on its protein levels. Ovarian tissue sections showed immunoreactivity with an antibody against this cyclase in granulosa cells (stars in Fig 6) as well as corpus luteal cells (CL in Fig 6). The signal intensity appeared greater in a mutant mouse 9 hours after exposure to male-derived scent (Fig 6D) than that seen in ovaries from either a wild type (Fig 6A) or a mutant (Fig 6C) unisexually isolated mouse not exposed to male derived scent, or from a wild type mouse exposed to such scent for 9 hours (Fig 6B). A more extensive study with a larger number of mice examined at different time points following exposure to male-derived scent is needed to more accurately characterize the differences in intra-ovarian adenylyl cyclase 3 expression between these different conditions. The results, nevertheless, suggest that there is an increase in protein levels of this cyclase following exposure to male-derived scent that appears to be more pronounced in mutant granulosa cells. While the possibility remains that this increase is due to activation of another G protein-coupled receptor because this cyclase does not respond exclusively to olfactory receptors, the data are compatible with the idea that absence of an active Brca1 protein in ovarian granulosa cells leads to increased olfactory receptor signaling in these cells.

## Discussion

Our results show that mice lacking a functional Brca1 protein in their ovarian granulosa cells respond more readily to male-derived olfactory stimuli than either their wild type counterparts, or mice that lack this protein in organs other than the ovary including the central nervous system. Triggering of the estrous cycle, the equivalent of the human menstrual cycle, is the specific response to male-derived olfactory stimuli that was measured in these studies. Although the exact function of olfactory receptors in the ovary is still unclear, these findings are intriguing in light of the well-established association between menstrual cycle activity and extra-uterine Müllerian cancer risk [2,3] and raise the possibility that increased responsiveness to olfactory stimuli, possibly sexual pheromones, in *BRCA1* mutation carriers may contribute, at least in part, to their elevated risk of familial breast and extra-uterine Müllerian carcinoma.

We used mice that carried a homozygous *Brca1* mutation in their ovarian granulosa cells in our experiments in order to maximize any measurable effect of such mutation in our experimental system. However, granulosa cells are not the site of origin of the cancers that typically develop in *BRCA1* mutation carriers [11]. Thus, these cells invariably carry a single mutant allele of *BRCA1* in such carriers, even in those who develop BRCA1-associated malignancies. We argued earlier that the consequences of a heterozygous *BRCA1* mutation on gene expression could be similar to those of a homozygous mutation, albeit of lesser magnitude, due to alterations in gene dosage [6]. We previously demonstrated that the levels of proteins associated with estradiol biosynthesis in granulosa cells of heterozygous mutant mice were indeed intermediate between those in wild type and those in homozygous mutant mice in support of this hypothesis [6]. Our current results demonstrate that this also applies to intra-ovarian olfactory receptor gene expression, at least at the RNA level.

Olfactory receptors, which are part of the G protein-coupled receptor family, are expressed in most organs [12]. Although there are still limited data about their function outside the central nervous system, they are thought to play a role in regulation of renin secretion and glomerular filtration rate [22]. They also activate members of the MAPK family and inhibit cell proliferation in the prostate [23]. Support for the idea that intra-ovarian olfactory receptors respond to ligands present in the environment comes from our finding that mice harboring *Brca1 mutations confined to the* ovaries responded quicker to environmental stimuli than mutant mice harboring wild type ovaries. This does not constitute an absolute proof that environmental ligands bind directly to ovarian olfactory receptors because the possibility remains that increased circulating levels of estradiol, which we previously showed to be present in mice carrying a *Brca1* mutation in their granulosa cells [5], by causing a general increase in estrogen receptor levels in multiple organs including in the central nervous system, were responsible for the more rapid response observed in mice with mutant ovaries. However, the fact that responsiveness to male-derived scent can also be enhanced by removing the ovaries from their normal anatomical location and transplanting them under the renal capsule suggests that circulating estradiol levels, which are not increased by such transplantation procedures, cannot be solely responsible for the more rapid response seen in mutation carriers. Presence of adenylyl cyclase 3, a well-established downstream effector of G protein-coupled receptor signaling, within ovarian granulosa cells, further supports the presence of intra-ovarian olfactory receptor signaling, although the possibility remains that such expression reflects the activity of another receptor because this cyclase does not respond exclusively to olfactory receptors.

Regardless of the exact nature of the chemical that directly interacts with the ovary to trigger the estrous cycle in response to male-derived scent (i.e., whether it is the environmental agent itself or an endogenous hormone), it seems probable that it reaches the ovary via the blood circulation as opposed to the nearest opening to the environment, which is the vagina. We came to this conclusion based on the observation that mice carrying ovaries transplanted under their renal capsule, regardless of *Brca1* mutational status, responded more rapidly to olfactory stimuli than mice with intact endogenous ovaries. While these procedures result in the ovary being placed further away from the vaginal opening and also outside the confines of the bursa, it is likely that they also result in increased exposure to circulating blood elements due to the disproportionately high fraction of the cardiac output received by the kidney compared to intact ovaries.

Neuronal olfactory receptors have a level of regulation ensuring that a single receptor species is expressed in each neuron [24–27]. It is currently not clear whether or not a similar level of regulation is present in ovarian granulosa cells. The extent of variation in the spectrum of expression of these receptors between different follicles is also unknown. We noted that immunoreactive olfactory receptor proteins are present not only on the cell surface, but also in the cytoplasm, suggesting an important role for the unfolded protein response in their regulation, as is the case with neuronal olfactory receptors [14].

We suggested earlier that the *BRCA1* mutation carrier state in humans, in spite of its association with increased cancer risk, might confer some phenotypic advantages such as reduced predisposition to bone fractures due to increased estrogen exposure [6]. Our results point to yet another potential benefit of the *BRCA1* mutation carrier state, that of increased responsiveness to olfactory stimuli possibly impacting fertility, which may have contributed to maintaining such mutations in the human gene pool. This carrier state appears to have negligible consequences on the fertility of modern women [28], in whom it may in fact be associated with a slightly earlier menopause [29]. However, the presence of germline *BRCA1* mutations had positive effects on overall fertility in the early twentieth century before the development of oral contraceptives [30].

Our results are also relevant to the debate regarding the potential role of pheromones in influencing human social behavior and physiological processes including reproductive functions. While it is well-known that volatile chemicals present in the urine and other bodily fluids function as pheromones in rodents, their relevance to humans has been questioned because while pheromones signal through the vomeronasal system in these animals [31,32], a similar system has not been identified in humans. However, the possibility that humans, like rodents, respond to sexual pheromones is supported by observations such as synchronous menstrual cycles in women living in communal housing, for example college dormitories [33], and evidence for a role of body odors in mate selection by females [34–37]. It is especially intriguing, in the context of our present findings, that a role for body odor in mate selection was only seen in women with functioning ovaries [34,37]. Our results raise the possibility of using olfactory stimuli to increase fertility of individuals with poor ovarian function.

The menstrual cycle, the equivalent of the estrous cycle in mice, is the most important risk factor for breast and serous extra-uterine Müllerian carcinomas. Interfering with this cycle is protective against such cancers even in individuals with hereditary predisposition including *BRCA1* mutation carriers. Our data, if applicable to humans, suggests that the menstrual cycle is especially sensitive to olfactory ligands in *BRCA1* mutation carriers. This raises the question of whether or not interference with specific olfactory agents could be an effective means of reducing cancer risk in young individuals with germline *BRCA1* mutations who wish to postpone risk-reducing surgery in order to preserve their fertility.

## Supporting Information

S1 ARRIVE Checklist (PDF)

S1 Table Genes showing more than 2-fold change in their expression levels in *BRCA1* knockout compared to wild type mice. (PDF)
