# Supplementary material for: Brca1 Mutations Enhance Mouse Reproductive Functions by Increasing Responsiveness to Male-Derived Scent
Source: PLoS One. 2015 Oct 21;10(10):e0139013. doi: 10.1371/journal.pone.0139013 (PMC4619541; doi:10.1371/journal.pone.0139013)
Supplement: S1 Table — (PDF) [file pone.0139013.s002.pdf]

~~Table S1~~

Genes showing more than ~~2 fold~~ change in their expression levels in ~~BRCA1 knock out~~ compared to wild-type mice

Sr. No	*Entrez Gene*	*Gene Symbol*	*Gene Title*	*Fold change 8 month old Mutant vs Wild Type*	*Fold change 11 month old Mutant vs Wild Type*
1	105387	Akr1c14	aldo-keto reductase family 1, member C14	-3.2275336	-2.89016
2	19716	Bex1	brain expressed gene 1	-3.2264135	-2.26933
3	100737	Dcun1d4	DCN1, defective in cullin neddylation 1	-2.5877016	-3.50483
4	246700	Defb19	defensin beta 19	-2.1647947	-2.56471
5	67420	Mlstd2	male sterility domain containing 2	-2.2659726	-2.31208
6	17263	Meg3	maternally expressed 3	-2.0458584	-2.14598
7	56524	Mpp6	membrane protein, palmitoylated 6 (MAGUK p55 subfamily member 6)	-3.0406427	-2.65126
8	27057	Ncoa4	nuclear receptor coactivator 4	-3.1985514	-2.18744
9	26432	Plod2	procollagen lysine, 2-oxoglutarate 5-dioxygenase 2	-2.8397145	-2.20844
10	12304	Pdia4	protein disulfide isomerase associated 4	-2.1295295	-2.05612
11	66648	5730494M16Rik	RIKEN cDNA 5730494M16 gene	-2.6381388	-2.12473
12	268591	Serpina5	serine (or cysteine) peptidase inhibitor, clade A, member 5	-3.8366585	-2.29611
13	56274	Stk3	serine/threonine kinase 3 (Ste20, yeast homolog)	-2.0507965	-2.21193
14	105727	Slc38a1	solute carrier family 38, member 1	-2.497845	-2.108
15	20230	Satb1	special AT-rich sequence binding protein 1	-2.0611615	-2.07698
16	245282	Apol10a	apolipoprotein L 10a	2.5542405	3.413476
17	278679	Apol7b	apolipoprotein L 7b	5.8377657	2.400769
18	108956	Apol7c	apolipoprotein L 7c	2.1527157	3.206917
19	77674	Defb12	defensin beta 12	3.1166632	2.411853
20	13215	Defb2	defensin beta 2	4.2127724	3.054842
21	13532	Dub2a	deubiquitinating enzyme 2a	5.3082666	7.008603
22	73693	Dppa4	developmental pluripotency associated 4	2.4576125	2.609262
23	386753	Dbpht2	DNA binding protein with his-thr domain	2.3305104	2.049873

24	13836	Epha2	Eph receptor A2	2.2074127	2.294999
25	15267	Hist2h2aa1	histone cluster 2	2.206948	2.097853
26	78303	Hist3h2ba	histone cluster 3, H2ba	2.4493928	2.652023
27	110350	Impg1	interphotoreceptor matrix proteoglycan 1	2.0143955	2.109646
28	16619	Klk1b24	kallikrein 1-related peptidase b27	2.1475165	2.010029
29	68720	Lce1b	late cornified envelope 1B	2.2632146	2.030189
30	66203	Lce1m	late cornified envelope 1M	3.212659	2.668287
31	667977	H2-t9	MHC class Ib T9	2.034055	2.142417
32	140474	Muc4	mucin 4	2.0578191	2.153998
33	17928	Myog	myogenin	2.1727376	2.513576
34	11924	Neurog2	neurogenin 2	2.2599025	2.27081
35	666532	RP24-320O9.1	novel KRAB box containing protein	2.4541018	3.296345
36	258218	Olfr102	olfactory receptor 102	3.4002094	3.375257
37	258573	Olfr1020	olfactory receptor 1020	2.9031682	2.167975
38	259015	Olfr1038	olfactory receptor 1038	2.751442	2.72862
39	258331	Olfr1338	olfactory receptor 1338	2.128158	2.152085
40	258383	Olfr1347	olfactory receptor 1347	2.1259787	4.68578
41	404337	Olfr1383	olfactory receptor 1383	6.2076635	5.795528
42	257959	Olfr144	olfactory receptor 144	3.5219624	2.75534
43	258678	Olfr1441	olfactory receptor 1441	2.4363356	2.482448
44	235256	Olfr149	olfactory receptor 149	5.14012	3.502697
45	258151	Olfr1505	olfactory receptor 1505	3.4783664	3.501614
46	57270	Olfr1508	olfactory receptor 1508	2.1119268	2.112735
47	57269	Olfr1507	olfactory receptor 1507	2.1964102	2.239952
48	258477	Olfr197	olfactory receptor 197	2.0960355	3.350093
49	258502	Olfr234	olfactory receptor 234	2.0168762	2.096754
50	258266	Olfr247	olfactory receptor 247	2.708436	3.175918
51	258304	Olfr498	olfactory receptor 498	3.735642	2.039516
52	259118	Olfr575	olfactory receptor 575	4.348001	2.165265
53	18363	Olfr62	olfactory receptor 62	3.9137306	2.005136
54	259075	Olfr641	olfactory receptor 641	2.8566837	2.934046
55	259100	Olfr666	olfactory receptor 666	4.7805824	3.350573
56	18369	Olfr68	olfactory receptor 68	3.5257833	3.5046818
57	258316	Olfr727	olfactory receptor 727	2.2776103	2.472018
58	258933	Olfr796	olfactory receptor 796	2.2261665	2.219317
59	258670	Olfr820	olfactory receptor 820	2.168449	2.394081
60	18511	Pax9	paired box gene 9	2.0545714	2.29848
61	19064	Ppy	pancreatic polypeptide	2.099507	2.619522
62	245841	Polr2h	polymerase (RNA) II (DNA directed) polypeptide H	2.0463183	2.61851
63	18768	Pkib	protein kinase inhibitor beta, cAMP dependent, testis specific	2.063318	2.020805

64	212108	Rln3	relaxin 3	2.0647671	2.424963
65	20088	Rps24	ribosomal protein S24	2.294455	2.059654
66	381393	4921509C19Rik	RIKEN cDNA 4921509C19 gene	15.48181	5.785097
67	442814	5031438A03Rik	RIKEN cDNA 5031438A03 gene	4.6938324	2.611066
68	319574	9330133O14Rik	RIKEN cDNA 9330133O14 gene	2.1998394	3.136568
69	319630	A130014H13Rik	RIKEN cDNA A130014H13 gene	5.019434	3.684361
70	109095	Rbm15b	RNA binding motif protein 15B	2.0727198	2.692324
71	20201	S100a8	S100 calcium binding protein A8 (calgranulin A)	5.829469	2.100187
72	20700	Serpina1d	serine (or cysteine) peptidase inhibitor, clade A, member 1d	2.5844655	3.205116
73	100042767	LOC100042767	similar to ribosomal protein L21	3.4977264	2.617244
74	20753	Sprr1a	small proline-rich protein 1A	3.6524174	2.41062
75	20755	Sprr2b	small proline-rich protein 2B	3.0058913	2.847626
76	229562	Sprr4	small proline-rich protein 4	3.9863122	2.705184
77	74673	4933411G11Rik	speedy homolog B (Drosophila)	2.2258465	2.119306
78	17235	Smcp	sperm mitochondria-associated cysteine-rich protein	3.178735	2.411969
79	21786	Tff3	trefoil factor 3, intestinal	2.0352447	2.539481
80	381058	Unc93a	unc-93 homolog A (C. elegans)	2.1979942	2.125627
81	22412	Wnt9b	wingless-type MMTV integration site 9B	2.17238	2.051607
82	20834	Znrf4	zinc and ring finger 4	3.6402836	2.35518
